# Dramatic response to modified docetaxel, cisplatin, and fluorouracil chemotherapy after immunotherapy in a patient with refractory metastatic anal cancer

**DOI:** 10.1002/ccr3.2333

**Published:** 2019-08-06

**Authors:** Faisal Shahjehan, Saivaishnavi Kamatham, Ashton Ritter, Pashtoon Murtaza Kasi

**Affiliations:** ^1^ Division of Hematology and Oncology Mayo Clinic Jacksonville FL USA; ^2^ College of Medicine and Oncology University of Iowa Iowa City IA USA

**Keywords:** chemotherapy, cisplatin, fluorouracil, immunotherapy, mDCF, metastatic anal cancer, modified docetaxel

## Abstract

Doublet chemotherapies are the mainstay in the management of metastatic anal cancer. Immunotherapy has been incorporated into guidelines in a refractory setting. Treatment options remain limited with tumor progression beyond immunotherapy. Modified docetaxel, cisplatin, and fluorouracil (mDCF) chemotherapy, after progression postimmunotherapy, has shown a near‐complete response in our patient with metastatic anal cancer. This likely is secondary to the sequence of immunotherapy followed by chemotherapy that is yielding greater than historical responses.

## 1 INTRODUCTION

For metastatic anal cancer, doublet chemotherapies are the mainstay in management with immunotherapy in the refractory setting. The options are limited for patients with postprogression on immunotherapy. We report a case of metastatic anal cancer that postprogression to immunotherapy showed a near‐complete response to modified docetaxel, cisplatin, and fluorouracil chemotherapy.

Anal cancer is a relatively rare disease that accounts for about 2.7% of all gastrointestinal cancers.^1^ However, its incidence has been increasing for the last few decades in both men and women.^2^ The majority of patients are diagnosed at an early‐stage with favorable outcomes with definitive chemoradiation being the mainstay. On the other hand, metastatic anal cancer is associated with poor prognosis with a 5‐year survival rate of 15% for squamous cell subtype as estimated by American Cancer Society.^3^ Systemic therapy options for patients with metastatic anal cancer are limited. Doublet chemotherapies in the form of fluoropyrimidine (5‐fluorouracil; 5‐FU)‐, platinum (Cisplatin, Carboplatin)‐, and/or Taxane (Paclitaxel)‐based therapies are the mainstay in management. Immunotherapy in the form of anti‐PD1 drugs (nivolumab/pembrolizumab) has also been incorporated into guidelines in the refractory setting. Postprogression on immunotherapy and upfront chemotherapy options are limited, responses poor, and duration limited. Recently, modified docetaxel, cisplatin, and fluorouracil (mDCF) regimen was shown to have excellent and durable activity in the first‐line setting with manageable adverse events.^4^

Here, we report a case of metastatic anal cancer that postprogression to immunotherapy nivolumab showed a near‐complete response with all pulmonary metastases (disappearance >20 lesions) and one liver lesion with more than 50% shrinkage with mDCF chemotherapy. This is in a patient with prior exposure to carboplatin, paclitaxel as well as 5‐FU/mitomycin upfront chemotherapy. The dramatic response may be secondary to prior exposure to immunotherapy, which is increasingly being reported across multiple tumor types where the sequence of immunotherapy followed by chemotherapy is yielding greater than historical responses^5^, ^6^, ^7^, ^8^, ^9^, ^10^ as summarized in Table 1.

**Table 1:** Studies reporting on the utility of chemotherapy after immunotherapy in various cancers

Authors	Year	Study type	Primary cancer (no. of patients)	Immunotherapy used	Chemotherapy used	Responses measured
Dwary et al^5^	2017	Case series	Metastatic head and neck cancer (3), non‐small cell lung cancer (2), T ‐cell rich B‐ cell lymphoma (1)	Pembrolizumab, nivolumab	Paclitaxel/carboplatin, docetaxel, gemcitabine	Complete response measured by PET CT scan
Park et al^9^	2018	Retrospective	Metastatic non‐small cell lung cancer (73)	Pembrolizumab, nivolumab, durvalumab, avelumab, tremelimumab, atezolizumab	Salvage chemotherapy administered after immunotherapy (SCAI)	Objective response rates (ORRs); ORR of patients treated with SCAI was 53.4% compared to ORR of 34.9% in patients with last chemotherapy administered before immunotherapy (LCBI)
Shiono et al^10^	2019	Retrospective	Non‐small cell lung cancer (20)	Nivolumab	Ramucirumab plus docetaxel	Overall response rate of ramucirumab plus paclitaxel was 60%, progression‐free survival was 169 d, and overall survival was 343 d.
Chakrabarti et al^7^	2018	Case series	Metastatic gastroesophageal adenocarcinoma (2)	Pembrolizumab	Paclitaxel	Complete response
Goldinger et al^8^	2018	Retrospective case series	Metastatic melanoma (463)	Ipilimumab, PD1 antibodies, combination immunotherapies	Carboplatin, paclitaxel, dacarbazine, temozolomide, taxanes, fotemustine	Objective response rate (ORR) of 11%, median progression‐free survival (PFS) of 2.5 mo. Best results were seen in 41 patients who received taxanes, with ORR of 27% and median PFS of 3.9 mo.

## 2 CASE PRESENTATION

Patient is a 59‐year‐old lady who initially presented with bright red blood per rectum. She had a past history of Bartholin cyst removal years ago and subsequently has had rectovaginal fistula with multiple repairs. Worsening pain prompted more workup and imaging that showed the pelvic mass. In January 2017, MRI was done at Mayo Clinic which showed a polypoid mass in the lower rectum/anus with extension through the anterior wall of the rectum. It was associated with enlarged left external iliac lymph nodes and enhancing mass on the left side of the pelvis which extended into the sciatic notch. She had a CT‐scan of the chest that revealed scattered bilateral sub centimeter pulmonary micro nodules which at the time were indeterminate. There was no prior dedicated prior chest imaging to compare the differences in some of these nodules.

Biopsy came back as poorly differentiated squamous cell cancer. After a multidisciplinary tumor board discussion and consensus, definitive chemoradiation with fluorouracil and mitomycin was pursued in February 2017 with excellent clinical response with improvement of pain/bleeding.

**Figure 1:**
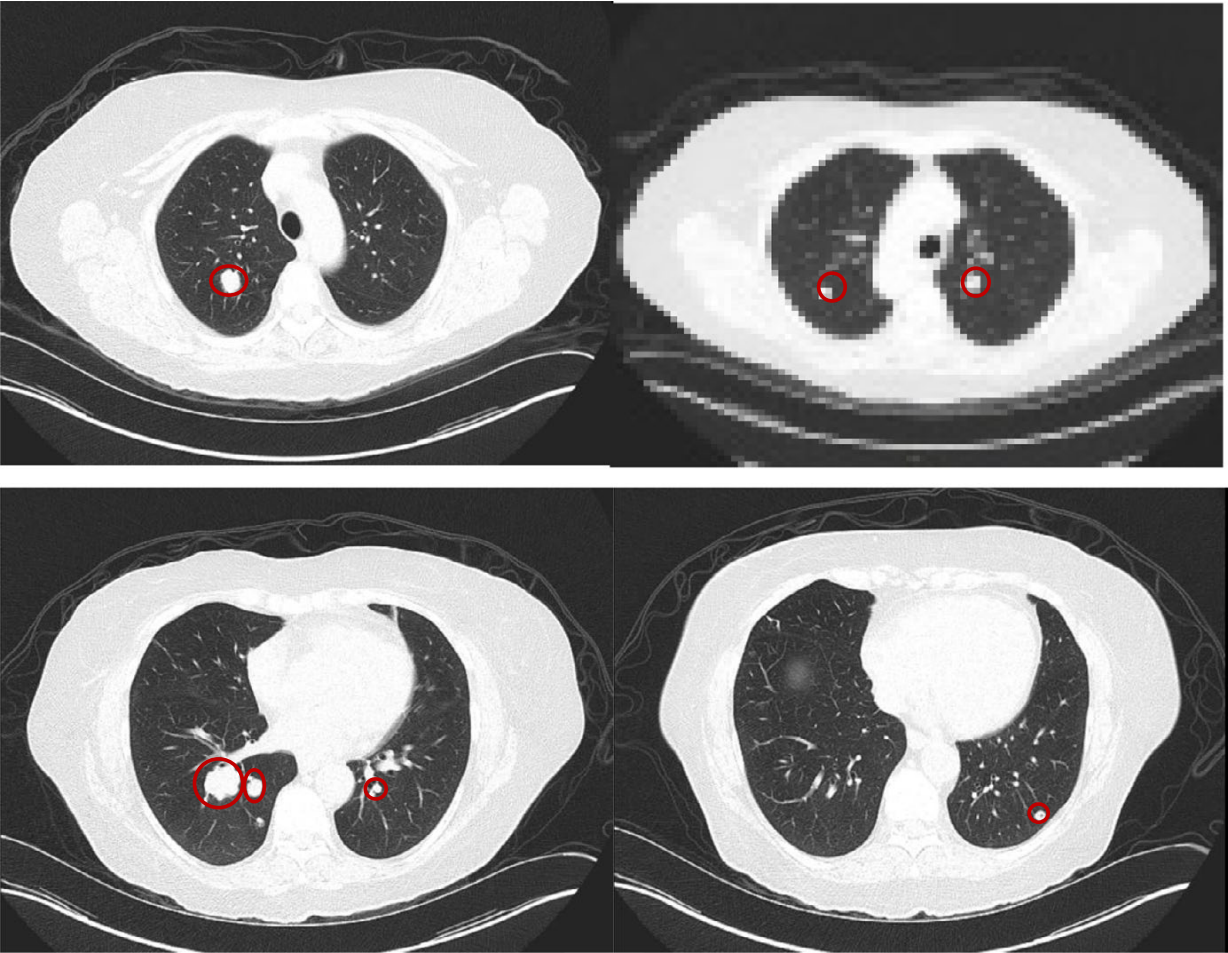
CT scans of the patient before modified‐ docetaxel, cisplatin, and fluorouracil (DCF) chemotherapy showing multiple lung metastases

**Figure 2:**
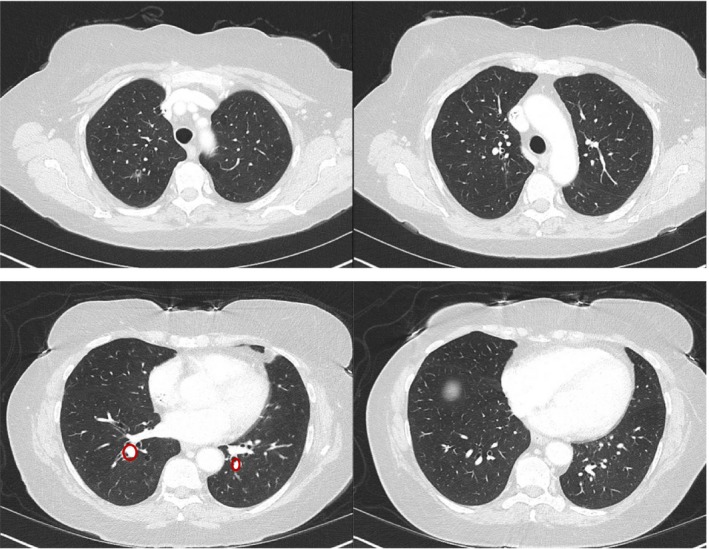
CT scans showing a near‐complete resolution of lung metastases after modified‐ docetaxel, cisplatin, and fluorouracil (DCF) chemotherapy

Scans in May 2017 marked shrinkage in anorectal mass, left pelvic sidewall mass and metastatic external iliac lymph nodes. There was no evidence of metastatic disease in the abdomen. However, enlargement in the previously observed lung nodules was noted. This was now amenable to a biopsy that showed metastatic poorly differentiated squamous cell carcinoma that was consistent with the patient's history of anal primary. There was normal expression of MLH1, MSH2, MSH6, and PMS2; and no expression of PD‐L1 (less than 1% of viable tumor cells exhibit membrane staining at any intensity). She was started on doublet chemotherapy with carboplatin and paclitaxel in July 2017. She had initial response followed by later progression noted in February 2018. She was switched to single agent nivolumab in February 2018 which she took till June 2018. Subsequent scans in June 2018 showed significant progression of disease (multiple >20 nodules, growth of existing lung metastases measuring on average more than 2 cm as well as a new liver lesion as measuring 1.6 cm). At the time, given the significant growth and lack of clinical trials but still excellent performance status of the patient, modified DCF (mDCF) was chosen given the data published by Kim et al in Lancet Oncology on 2 July 2018. We repeated the imaging after 2 months of treatment in September 2018, which showed a near‐complete ongoing response (>20 lung lesions now indiscernible; liver mass now less than 1 cm). The CT scans done pre‐DCF therapy and those done post‐DCF therapy are shown in Figures 1 and 2. The dramatic response may be secondary to prior exposure to immunotherapy, which is increasingly being reported across multiple tumor types where the sequence of immunotherapy followed by chemotherapy is yielding greater than historical responses.

## 3 DISCUSSION

Refractory metastatic anal cancer postprogression on chemotherapy and immunotherapy have limited options.^11^ Approximately 10%‐20% of patients present with metastatic anal cancer.^12^, ^13^, ^14^ The most common sites of metastasis from anal cancer include lungs, liver, and bones.^15^

Previous studies have demonstrated a variety of risk factors responsible for the development of anal cancer. Immunocompromised status secondary to the use of immunosuppressive agents, receiving solid organ transplantation, or any systemic illness like Crohn's disease increases the risk of anal cancer.^16^, ^17^, ^18^, ^19^ High‐risk sexual behavior with the involvement of carcinogenic human papillomavirus infection (HPV) is also an established risk factor of the disease.^20^ Similarly, human immunodeficiency virus (HIV) infection also increases the anal cancer risk especially in HIV‐infected men who have sex with men.^21^ Cigarette smoking has also been reported to be associated with anal cancer however the role of other confounders for example unsafe sexual practices cannot be excluded.^22^, ^23^ A case‐control study of 306 anal cancer patients and 1700 control participants reported that the current smoking status increases the risk of anal cancer in both men (OR: 3.9; 95% CI: 1.9‐8.0) and women (OR:3.8;95% CI: 2.4‐6.2).^24^ Additionally, mutations in tumor suppressor genes, for example, TP53 play a role but no relevant actionable drivers.^25^

For metastatic cancer, the main treatment modalities for metastatic anal cancer include systemic chemotherapy and immunotherapy. The current NCCN (National Comprehensive Cancer Network) guidelines recommend three chemotherapy drug classes in different combination regimens for the treatment of metastatic anal cancer. These include (a) Fluoropyrimidines [5‐fluorouracil (5‐FU), capecitabine] (b) Platinum drugs (cisplatin, carboplatin, oxaliplatin) and (c) Taxanes (paclitaxel, docetaxel). Several studies have illustrated the efficacy of these chemotherapy drugs in the management of metastatic anal cancer.^26^ The combination regimen with 5‐FU and cisplatin has been a well‐recognized treatment for metastatic anal cancer as manifested from several previous studies.^27^, ^28^, ^29^ More recently, Rao et al revealed the results of a randomized controlled trial comparing 5‐FU/Cisplatin‐based therapy with Carboplatin/Paclitaxel at the European Society of Medical Oncology (ESMO) 2018 meeting (InterAACT Study). The study included inoperable locally recurrent or metastatic anal cancer patients (n = 91) from four countries including UK, Norway, United States, and Australia. Their results showed that Carboplatin/Paclitaxel was associated with better response rates (59% vs 57.1%), increased median OS (20 vs 12.3 months), and lesser SAEs (36% vs 62% of patients) compared to 5‐FU/Cisplatin.^30^ The carboplatin with paclitaxel is now considered the preferred first‐line regimen based on discussions pertaining to the report. Additionally, however, in July 2018, Kim et al reported the results, published in Lancet Oncology, of a phase 2, multicenter clinical trial to demonstrate the effects of DCF chemotherapy for patients with metastatic or unresectable locally recurrent anal cancer. They included 66 patients of whom 36 received standard DCF regimen and 30 received dose‐modified DCF regimen. Their results indicated that the modified DCF regimen (docetaxel 40 mg/m^2^ and cisplatin 40 mg/m^2^ on day 1 and fluorouracil 2400 mg/m^2^ per day over 46 hours biweekly) was associated with better tolerability and less SAEs compared to standard DCF regimen. The study reported dramatic responses including complete responses in their cohort, with 47% patients were progression‐free and alive at 12 months.^4^

Immunotherapy with immune‐checkpoint inhibitors are also studied and an area of ongoing research for the treatment of metastatic anal cancer. Among those, the anti‐programmed cell death‐1 (anti‐PD1) antibodies including nivolumab and pembrolizumab are shown to be effective agents against the metastatic anal cancer and incorporated in the guidelines in the refractory setting. Morris et al conducted a phase 2 trial of metastatic squamous cell‐subtype anal cancer patients (n = 37) and evaluated the efficacy of nivolumab. Their results showed an objective response rate and a disease control rate of 24% and 72%, respectively. The median progression‐free survival was 4.1 months, 6‐month progression‐free survival of 38%, median overall survival of 11.5 months, and 1‐year overall survival of 48%.^31^ Similarly, Ott et al did a phase 1b trial (KEYNOTE‐028 study) studying the safety and efficacy of pembrolizumab in metastatic anal cancer patients (n = 25) who were programmed death ligand 1 (PD‐L1)‐positive. The researchers reported that 10 (42%) patients had stable disease and their cohort had an overall response rate of 17%. However, 64% of the enrolled patients had adverse effects secondary to pembrolizumab.^32^ Despite this, not many patients respond to these therapies and the results are not always durable. Our patient as well did not respond to nivolumab and had significant progression warranting us to consider the mDCF regimen. As noted, patient had a near‐complete response with all pulmonary metastases (disappearance > 20 lesions) and one liver lesion with more than 50% shrinkage. This is in a patient with prior exposure to carboplatin, paclitaxel as well as 5‐FU/mitomycin upfront chemotherapy. The dramatic response may be secondary to prior exposure to immunotherapy, which is increasingly being reported across multiple tumor types where the sequence of immunotherapy followed by chemotherapy is yielding greater than historical responses. The limitation of this is firstly this is a case report. Secondly, we at present do not completely understand the mechanisms to why the sequence potentially may be a novel mechanism. The combination of immunotherapy and chemotherapy and/or biologics is being studied in multiple clinical trials.

## CONFLICT OF INTEREST

Consulting/Advisory board: PMK (Ipsen/Taiho to institution). Research Funding (To institution)—Advanced Accelerator Applications; Array BioPharma; Bristol‐Myers Squibb; Celgene. No other conflicts of interests to report for the authors.

## AUTHOR CONTRIBUTIONS

PMK instigated, guided, and revised the manuscript. FS wrote the manuscript. AR and SK provided the case details and CT scans and revised the manuscript. All authors approved and edited the final version before publication.
